# Outcome switching in randomized controlled oncology trials reporting on surrogate endpoints: a cross-sectional analysis

**DOI:** 10.1038/s41598-017-09553-y

**Published:** 2017-08-23

**Authors:** Alberto Falk Delgado, Anna Falk Delgado

**Affiliations:** 1 Department of Surgical Sciences, Uppsala University, Uppsala, Sweden; 2 Clinical Neuroscience, Karolinska Institute, Stockholm, Sweden; 3 Department of Neuroradiology, Karolinska University Hospital, Stockholm, Sweden

## Abstract

Inconsistent reporting of clinical trials is well-known in the literature. Despite this, factors associated with poor practice such as outcome switching in clinical trials are poorly understood. We performed a cross-sectional analysis to evaluate the prevalence of, and the factors associated with outcome switching. PubMed and Embase were searched for pharmaceutical randomized controlled trials (RCTs) in oncology reporting on a surrogate primary outcome published in 2015. Outcome switching was present in 18% (39/216). First-author male sex was significantly more likely associated with outcome switching compared to female sex with an OR of 3.05 (95% CI 1.07–8.64, *p* = 0.04) after multivariable adjustment. For-profit funded RCTs were less likely associated with outcome switching compared to non-profit funded research with an OR of 0.22 (95% CI 0.07–0.74, *p* = 0.01). First author male sex was more likely associated with outcome switching compared to female sex in drug oncology RCTs reporting on a primary surrogate endpoint. For-profit funded research was less likely associated with outcome switching compared to research funded by non-profit organizations. Furthermore, 18 percent of drug oncology trials reporting on a surrogate endpoint could have a higher risk of false positive results due to primary outcome switching.

## Introduction

Selective and inconsistent reporting of randomized controlled trials (RCTs) is well-known in the literature^1, 2^. Outcome switching is one type of inconsistent reporting in RCTs. A shortage of pre-specified analysis and associated power estimation can lead to false positive results and lack of reproducibility^2^. The development of clinical trial registration is one measure to increase transparency in the reporting of RCTs. Protocol registration might reduce discrepancies between registered trial protocol and published manuscript such as outcome switching^3^. Mandatory clinical trial registration was implemented in 2005 by the International Committee of Medical Journal Editors^4^.

In a study by Jones *et al*. in 2015^5^, the investigators found a discrepancy between registered primary outcome and published primary outcome in a median of 31% of included studies. Despite previous efforts, specific factors associated with outcome switching in published randomized trials are still poorly understood^5, 6^.

The usage of surrogate endpoints such as progression-free survival (PFS) or biochemical results are cumbersome, since they are related to a lack of clear or direct clinical benefit for the patient and does not necessarily improv clinical outcome or overall survival^7–9^. The translation of a surrogate endpoint to a clinical benefit for the patient is problematic since the outcome might in itself lack a clinical effect^10^. Unfortunately, acknowledgement of the usage of surrogate endpoint usage is only disclosed in approximately one third of RCTs reporting on surrogate endpoints^10^. In addition, the usage of primary surrogate endpoints is widely spread among oncology RCTs. The usage of surrogate outcome is attractive since this speeds up the process of developing new drugs. This explains why there is an increasing trend for selecting surrogate endpoints as the primary outcome, while overall survival as a primary endpoint is decreasing^11^. Cancer drug approval by the FDA (Food and Drug Administration) that is exclusively based on trials with a primary surrogate endpoint has been reported to 74% (39/53)^12^. At worst, a study affected by outcome switching to a poorly validated surrogate endpoint might result in problems with trial reproducibility and limited clinical effect.

We hypothesized that outcome switching still occurs in oncology drug trials reporting on surrogate endpoints^13^ despite mandatory trial registration. Further, we hypothesized that outcome switching would be associated with specific study characteristics.

The primary aim of this study was to assess the prevalence of, and the factors associated with primary outcome switching among oncology trials reporting on a surrogate outcome.

## Methods

### Search strategy

A librarian conducted a search for randomized clinical trials in oncology published in 2015, using controlled vocabulary for more precision. The search was performed in PubMed and Embase. A full documentation of the search strategy is presented in Supplementary Tables 1–2. Primary drug studies reporting on a surrogate primary outcome were selected for inclusion by one author (Al.F.D.). Only studies reporting on anti-cancer drugs were eligible for inclusion. Focus on the primary outcome was chosen since it is used for sample size calculation and represent the most relevant outcome. A surrogate endpoint was defined as an endpoint with an unclear consequence for the patients’ overall survival for example; progression free survival, relapse free survival, response rate, biochemical testing or imaging.

Exclusion criteria were: meta-analyses, follow-up studies, phase-1 trials, abstracts, reviews, non-anticancer treatment such as nausea or pain medication, and non-pharmaceutical treatment such as surgery. The study was conducted according to the PRISMA guidelines^14^.

Included studies were evaluated in full text to search for a reference to a registered protocol. The date at which the protocol was first registered was compared to the date for initiation of patient recruitment by one investigator (An.F.D.). Initiation of patient recruitment before protocol registration was classified as retrospective protocol registration. Protocol registration before patient recruitment was classified as prospective protocol registration.

### Switching categorization

Outcome switching was independently assessed and extracted by two authors [Al.F.D, An.F.D.] through comparison of the original trial protocol in public databases with the published manuscript. Further, the presence of switching was defined as minor or major according to a modified version of Chan *et al*.^3^ described in Table 1. This definition also describes minor switches (reformulated pre-specified outcomes or rearrangements in pre-specified lists of several primary outcomes).

**Table 1:** Definition of switching.

Type of switch	Switching from original outcome	Primary outcome in trial protocol	Primary outcome in manuscript
No Switch	No switching	One primary outcome	Same primary outcome
Minor switching	Minor switching	Several primary outcome	One primary outcome chosen
	Minor switching	Several general outcome	Primary outcome the first in general list
	Minor switching	One primary outcome	Reformulated primary outcome
	Minor switching	Several general outcome	Primary outcome not first in general list
Major switching	Major switching	No primary outcome	New primary outcome
	Major switching	Primary outcome reported	Initially secondary outcome as primary outcome
	Major switching	Primary and secondary outcome reported	Primary outcome not congruent with either primary or secondary outcome

### Data extraction

For each trial, pre-specified data was tabulated independently by two authors including: funding source (for-profit/non-profit/mixed), first author sex (male/female), conflict of interest (COI) (yes/no), control group (active/placebo/observation), journal impact factor (defined 2015, continuous data), number of randomized patients and type of primary surrogate endpoint (continuous data). This data was tabulated blinded for information on switching.

Funding source was based on statements in published manuscripts. First author sex was determined by inspection of the authors first name, with electronic search performed if necessary as previously described^15^. Two investigators [Al.F.D., An.F.D.] classified names as female or male sex independently with results checked for congruency.

### Statistical analysis

Descriptive data was described using medians and interquartile range, or absolute number and percentage. Data was stratified for switching or no switching. Pre-specified study characteristics and their association with outcome switching was evaluated with univariable and multivariable analysis. A generalized logit model with binominal distribution of the dependent variable and sigma-restricted parameterization with a maximum of 100 iterations was built to test for all effects. We decided a priori to keep all variables from the univariable analysis in the multivariable model. In a sensitivity analysis we kept variables with *p* < 0.1–0.2. In the multivariable analysis the between effects of all included study characteristics were evaluated. Unadjusted and adjusted odds ratio (OR) were reported with 95% confidence intervals. Interactions in the model were explored. Interaction terms with dummy variables were used to test for significant interactions in the logistic regression model. The goodness of fit in the logistic regression model was evaluated with the Hosmer-Lemeshow test. All *p*-values were two-sided. Statistical analyses were performed in Statistica v12 and SPSS v 20.

This study did not require an ethical approval since it only included previously published data.

## Results

### Selection process

**Figure 1:**
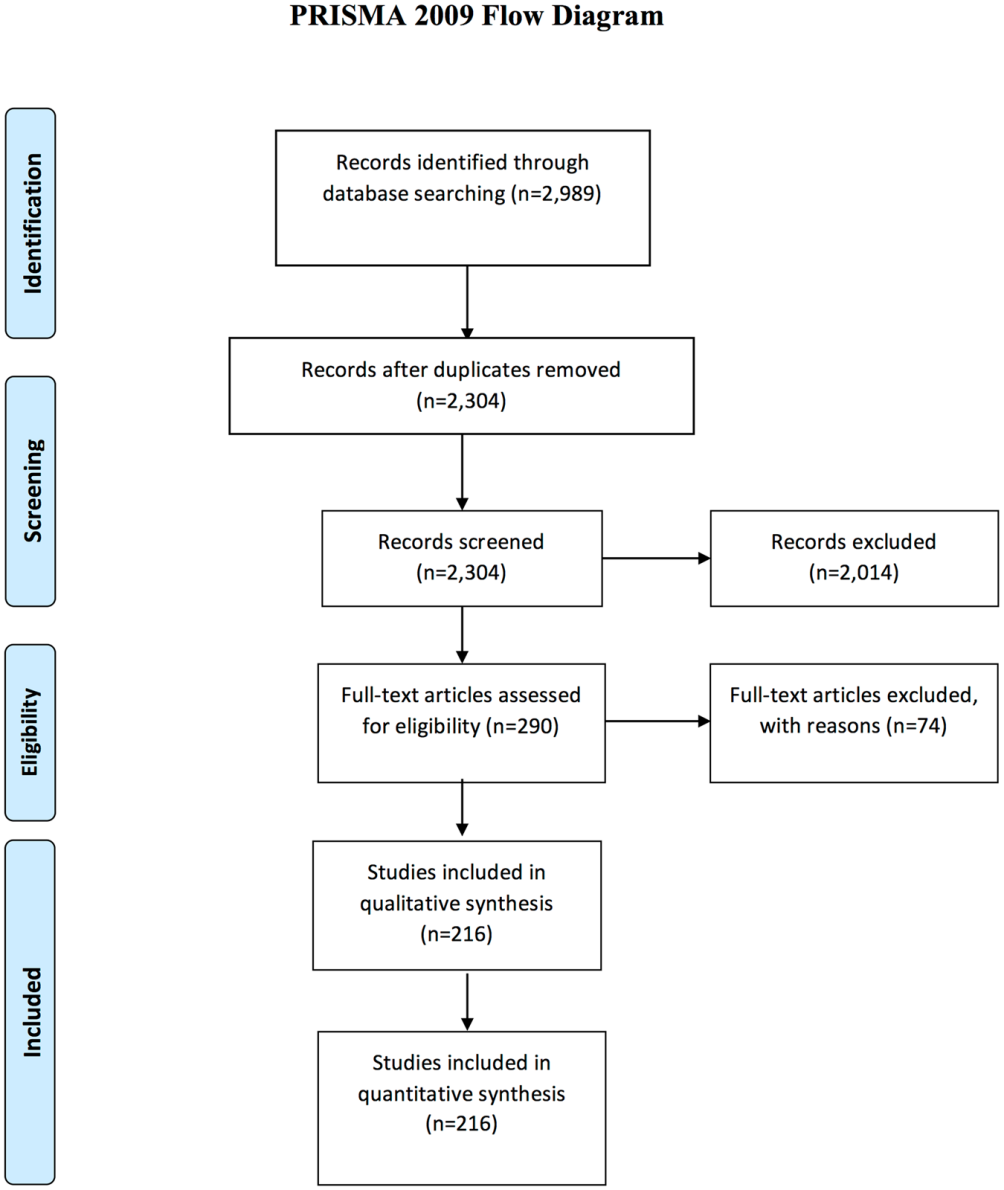
PRISMA Flow Chart.

The search yielded 2,989 hits. After removal of duplicates 2,304 were screened for possible inclusion. Full-text evaluation was performed in 290 with 26% (74/290) excluded due to lack of trial registration. A PRISMA flow chart summarizing the selection process is presented in Fig. 1.

### Characteristics of included studies

Quantitative analysis included 216 randomized controlled trial. Supplementary Table 3 shows details of the included studies. Characteristics of the included studies stratified for switching are shown in Table 2. Prospective protocol registration was present in 64% (n = 138) with retrospective protocol registration present in 36% (n = 78) of included studies.

**Table 2:** Characteristics of included studies.

Impact factor (categorized)	All studies (n = 216)	Studies with switching (n = 39)	Studies without switching (n = 177)
	Number (%)	Number (%)	Number (%)
High (>25)	20 (9)	3 (8)	17 (10)
Intermediate (≥15 ≤ 25)	72 (33)	16 (41)	56 (32)
Low (<15)	124 (57)	20 (51)	104 (59)
**Sex**			
Male	165 (76)	34 (87)	131 (74)
Female	51 (24)	5 (13)	46 (26)
**Funding**			
For-profit	112 (52)	11 (28)	101 (57)
Mixed	49 (23)	12 (31)	37 (21)
Non-profit	41 (19)	9 (23)	32 (18)
NA	14 (6)	7 (18)	7 (4)
**Conflict of Interest**			
Yes	142 (66)	22 (56)	120 (68)
No	63 (29)	12 (31)	51 (29)
NA	11 (5%)	5 (13)	6 (3)
**Control group**			
Active	172 (80)	31 (80)	141 (80)
Placebo	34 (16)	4 (10)	30 (17)
Observation	10 (4)	4 (10)	6 (3)
**Type of primary surrogate outcome in published manuscript**			
Complete response/Overall response/Response rate	48 (22)	7 (18)	41 (23)
Progression free survival	101 (47)	13 (33)	88 (50)
Other	67 (31)	19 (49)	48 (27)
**Population**	**Median (IQR)**	**Median (IQR)**	**Median (IQR)**
Patients	171 (90–409)	159 (80–452)	173 (95–396)

In total, outcome switching was present in 18% (39/216) of all randomized controlled trials included in the study. The frequency of primary outcome switching was 24% (19/78) in retrospectively registered protocols and 14% (20/138) in prospectively registered protocols (Chi^2^ *p* = 0.06). Twenty studies with major switching and 19 studies with minor switching were observed. Examples of major switches according to Chan *et al*.^3^ was the presence of a new primary outcome or an initially secondary outcome listed as a primary outcome in the published manuscript. Examples of minor switches were related to reformulated primary outcomes or rearrangements in pre-specified lists of several primary outcomes.

In studies with outcome switching 28% (11/39) were funded by for-profit organizations. In studies lacking switching, for-profit funding was present in 57% (101/177). A first author female sex was observed in 13% (5/39) of RCTs with switching. In studies without switching 26% (46/177) of the first authors were females. The intraobserver reproducibility for sex classification was excellent with a kappa coefficient of 1.00.

### Outcome switching and association with study factors

Univariable binomial logistic regression analysis.

Univariable binomial logistic regression showed that for-profit funded research was significantly less likely associated with outcome switching presenting an OR (odds ratio) of 0.27 (95% CI 0.11–0.62, *p* = 0.002), Table 3.

**Table 3:** Association of outcome switch and study characteristics, after univariable and multivariable adjustments (binomial logistic regression).

Characteristics	Level of effect	Unadjusted OR for outcome switch (95% CI)	*p*-value	Adjusted OR for outcome switch (95% CI)	*p*-value
Study population	Number (continuous data)	1.00 (1.00–1.00)	0.05	1.00 (1.00–1.00)	0.08
Impact factor	Number (continuous data)	1.00 (0.98–1.02)	0.93	1.00 (0.97–1.03)	0.76
First author sex:	Female	Reference		Reference	
	Male	2.39 (0.88–6.47)	0.09	3.05 (1.07–8.64)	0.04
Control group	Whole effect		0.15		0.34
	Observation	Reference		Reference	
	Active	0.33 (0.09–1.24)	0.51	0.36 (0.08–1.51)	0.39
	Placebo	0.20 (0.04–1.03)	0.09	0.30 (0.05–1.73)	0.30
COI (any author)	No	Reference		1.00	
	Yes	0.61 (0.30–1.25)	0.18	1.22 (0.41–3.65)	0.72
Funding	Whole effect		0.01		0.03
	Non-profit	Reference		Reference	
	Mixed	0.79 (0.33–1.89)	0.28	0.58 (0.18–1.85)	0.65
	For-profit	0.27 (0.11–0.62)	0.002	0.22 (0.07–0.74)	0.01

### Multivariable binomial logistic regression analysis

We decided a priori to keep all the factors in the logistic regression model due to a possible association with outcome switching. Sensitivity analysis was performed by keeping factors with a *p*-value <0.1–0.2 from the univariable logistic regression analysis. Multivariable binomial logistic analysis showed that studies with a first author male sex and studies with for-profit funding were significantly associated with outcome switching after multivariable adjustment. First-author male sex was significantly more likely associated with outcome switching compared to female sex with an OR of 3.05 (95% CI 1.07–8.64, *p* = 0.04, Table 3). For-profit funded research was less likely associated with outcome switching compared to non-profit funded research with an OR of 0.22 (95% CI 0.07–0.74, *p* = 0.01). The Hosmer-Lemeshow goodness-of-fit test for the model gave *p* = 0.92, indicating evidence of a good fit in the model. No interactions were detected in the model. To assess for the robustness of the model we carried out sensitivity analyses that did not alter the model.

## Discussion

### Main findings

In this study, trial registration was available in 74% of drug oncology RCTs reporting on a primary surrogate endpoint published in 2015. Despite clinical trial registration, outcome switching still exists in 18% of registered RCTs (51% major; 49% minor switching). To our knowledge, this is the first study to observe a positive association between outcome switching and first-author male sex, and a negative association between switching and for-profit funded research.

### Strengths and weaknesses of the study

The strengths of this study are pertinent to the fact that we aimed to include all oncology drug RCTs published in 2015 reporting on a primary surrogate outcome. Further, we adopted a modified version of Chan *et al*.^3^ also defining minor switching between trial protocol and published RCT. This method was implemented to increase the awareness of, and to more easily communicate the extent of outcome switching in published RCTs^16^.

Data on study characteristics was extracted blinded for switching information, and thereby minimizing the risk of perception bias. Only one researcher screened all titles, but any ambiguities were solved through discussion among the authors. Both authors assessed all potential outcome switches and extracted data independently. A potential limitation to this study is that it only covers one medical field and that it only includes studies reporting on a primary surrogate endpoint. Thereby the generalizability outside this field is unclear. However, we find it unlikely that factors associated with outcome switching should differ significantly between medical fields. The confidence intervals are wide but significant, reflecting some uncertainty around the measured, although significant effect. We acknowledge that some trials are registered without mentioning this in the manuscript. Since studies not mentioning registration were not included in our study this is a potential limitation. A further limitation of our study is the reliability of sex assessment. Despite there was an excellent intraobserver reproducibility we cannot rule out the possibility that some of the authors sex have been misclassified.

### Strengths and weaknesses in relation to other studies

A strength of our study compared to similar studies are related to our inclusion criteria which are not restricted to high-impact journals. Certain similar studies have defined search criteria based on journal impact levels^16, 17^. Selecting only results from high-impact journals might bias results towards certain journals^18^.

In this study we reported a trial registration rate of 76%, which is slightly lower than previously reported 82% in high impact oncology journals 2009^17^. Outcome switching was noted in 18% of registered studies. In a study by Mathieu *et al*. including RCTs from higher impact journals, outcomes switching and inconsistent reporting of the primary outcome was noted in 31%^19^. A similar switching rate has been recently reported by Jones *et al*.^5^ The lower incidence of switching in our study could be attributed to a more stringent reporting in 2015 compared to 2008. Other differences between our study and that performed by Mathieu *et al*. are relatable to discrepancies in medical fields and journals. Outcome switching has further been reported in more recent publications restricted to high-impact journals^16^ and random samples of trials registered in clinicaltrials.gov^20^.

In the present study, 28% (11/39) of outcome switching altered a clinical endpoint to a surrogate endpoint. There is to our knowledge no previous study related to surrogate endpoint switching. Outcome switching to significant results from a non-significant result has been favored in a subset of included trials assessed in a study by Mathieu *et al*.^19^, however, no statistical test was performed to support this association. A survey sending out questionnaires to trialists’ has supported switching from non-significant to significant findings^3^. In a small study including 51 studies from major surgical journals, there was no difference in outcome switching related to funding source^9^.

Factors associated with discrepancies between trial protocol and published RCTs are poorly understood^5, 21^. There is to our knowledge no previous study assessing the role of first author sex and its association with outcome switching. Nor is there any study reporting on a significant association between funding source and outcome switching^5^.

### Meaning of the study

For-profit funded research was less likely associated with outcome switching. The negative association of switching and for-profit funding can possibly be explained by superior study design in for-profit funded trials. Well prepared industry funded trials, suggests a higher standard and more robust results. Considering that for-profit funded research often are conducted by professional CROs (Contract Research Organizations)^22^ it is probable that industry funded research is more well planned at the time of trial registration. However, for-profit funded research has also been associated with non-publication of trials and publication bias favoring positive results. Hence, the negative association between for-profit RCTs and outcome switching could be due to publication bias rather than superior study quality^23^. In this study, an association between outcome switching and first author male sex was found. Previous research has shown that males are overrepresented in scientific misconduct^24^. Also, women have a lower likelihood to undertake risky behavior^25^. Outcome switching towards positive results might overestimate treatment effects for published data.

### Policy implications

Primary surrogate endpoints are common in oncology RCTs. The primary reason for surrogate endpoint usage is a more rapid completion of trials^11^. Surrogate endpoints have a low correlation with survival. Manuscripts using surrogate endpoints have a low rate of acknowledging the usage of surrogate endpoints^13, 26^. FDA approval of oncologic drugs are mainly based on studies using primary surrogate endpoints^12^. By this it can be concluded that the usage of surrogate endpoints in oncology trials are problematic. Our findings of 18% prevalence of switching to primary surrogate outcome, indicates that 18% of similar studies have an increased probability of false positive results. Further, switching to a surrogate outcome from a clinical outcome increases the probability that the drug might lack a clinically relevant effect.

Even though improvement of outcome reporting does not protect against outcome switching, a few situations can be avoided by honest reporting. Recently, the U.S. Department of Health and Human Services issued the Final Rule, to clarify the submission of clinical trial registration and making the results accessible in a timely manner^27^. The Final rule includes specification of the outcome measure, in order to easier detect protocol violations. In our study we noted that many of the minor switches could have been minimized by a better quality control from Clinicaltrials.gov, which should request a clarification of the trial registration form when the primary outcome measure is reported in an unclear manner with for example several general aims or no primary outcome specified. Trial protocols are often mandatory with the submission of manuscript in high impact journals. We acknowledge that some secondary outcomes might be removed in the final manuscript. However, switching of the primary outcome is a potential severe violation to the whole trial and should be clearly stated in the manuscript if occurring. A clear statement of primary outcome switching helps the reader to interpret the article in its right context. National medical agencies responsible for drug approval such as the FDA and EMA should be cautious when considering oncology trials with a primary surrogate endpoint. A relevant clinical effect should be assured and results should be compared with the original trial registration. As previously have been suggested, the editors play a central role in comparing manuscripts with trial registration, since outcome switching appears to persist despite several measures to reduce it.

### Future perspectives

Future research should verify the findings of first author sex and funding source and their association with outcome switching in other medical fields. It is also unclear if the Final Rule will decrease the proportion of studies with outcome switching.

## Conclusion

Eighteen percent of drug oncology trials reporting on a surrogate endpoint could have a higher risk of false positive results due to primary outcome switching. Furthermore, the usage of surrogate endpoints might in itself be an inaccurate measure lacking clinically relevant effect. First author male sex is more likely associated with outcome switching compared to female sex. For-profit funded research is less likely associated with outcome switching compared to research funded by non-profit organizations.

## Electronic supplementary material

Supplementary Table 1-3
