# Supplementary material for: Outcome switching in randomized controlled oncology trials reporting on surrogate endpoints: a cross-sectional analysis
Source: Sci Rep. 2017 Aug 23;7:9206. doi: 10.1038/s41598-017-09553-y (PMC5569019; doi:10.1038/s41598-017-09553-y)
Supplement: Supplementary file 1 — Supplementary Table 1-3 [file 41598_2017_9553_MOESM1_ESM.doc]

**Outcome switching in randomized controlled oncology trials reporting on surrogate endpoints: a cross-sectional analysis**

Alberto F. Delgado, MD, MSc, PhD^1^, Anna F. Delgado, MD, PhD^2^,

^1^Department of Surgical Sciences, Uppsala University, Uppsala, Sweden

^2^Clinical Neuroscience, Karolinska Institute, Stockholm, Sweden; Department of Neuroradiology, Karolinska University Hospital, Stockholm, Sweden

Supplementary Table 1

**PubMed search**

| Date of Search: 2016-06-20  Number of hits: 1,309  Comments: Cancer | Field labels: |
| --- | --- |
| "Neoplasms/drug therapy"[Mesh Terms]  OR  (Neoplasms[MeSH Terms] AND Drug Therapy[MeSH Terms])  OR  Antineoplastic Agents[MeSH Terms]  Filters: Randomized Controlled Trial; Publication date from 2015/01/01 to 2015/12/31 | |

Supplementary Table 2

**Embase search**

| Date of Search: 2016-06-20  Number of hits: 1,680  Comments: Cancer | Field labels: |
| --- | --- |
| **No.**  **Query**  **Results**  **1,680**  **#6**  **#5** AND (**'article'**/it OR **'article in press'**/it OR **'letter'**/it OR **'note'**/it OR **'review'**/it OR **'short survey'**/it)  **1,977**  **#5**  **#3** AND **2015**:py AND [randomized controlled trial]/lim  **84,500**  **#4**  **#3** AND **2015**:py  **1,281,943**  **#3**  **#1** OR **#2**  **840,375**  **#2**  **'antineoplastic agent'**/exp/mj  **663,910**  **#1**  **'neoplasm'**/exp AND **'drug therapy'**/exp | |

| Supplementary Table 3. Details of included studies (n = 216) | | | | | | | | |
| --- | --- | --- | --- | --- | --- | --- | --- | --- |
| Study | Randomized population (n) | Control group (Active/Placebo/Observation) | Funding (For-profit/Non-profit/Mixed) | COI (Y/N) | Journal Impact factor 2015 | Switching (Yes/No) | Switching type (None/Minor/Major) | First author sex (Male/Female) |
| 1 | 182 | Active | For-profit | Y | 9 | No | None | Male |
| 2 | 216 | Active | For-profit | Y | 9 | No | None | Female |
| 3 | 46 | Active | Mixed | N | 5 | No | None | Female |
| 4 | 276 | Placebo | For-profit | Y | 25 | No | None | Male |
| 5 | 973 | Placebo | For-profit | Y | 21 | No | None | Female |
| 6 | 951 | Placebo | For-profit | Y | 25 | No | None | Male |
| 7 | 3006 | Active | Mixed | Y | 60 | Yes | Minor | Female |
| 8 | 1114 | Active | For-profit | Y | 25 | No | None | Male |
| 9 | 121 | Active | For-profit | Y | 25 | No | None | Male |
| 10 | 795 | Active | For-profit | Y | 25 | No | None | Male |
| 11 | 483 | Active | For-profit | Y | 25 | No | None | Male |
| 12 | 2980 | Active | Mixed | Y | 44 | No | None | Male |
| 13 | 3104 | Active | Mixed | Y | 44 | Yes | Minor | Male |
| 14 | 412 | Active | Mixed | Y | 25 | Yes | Minor | Male |
| 15 | 235 | Active | Non-profit | N | 25 | Yes | Minor | Male |
| 16 | 222 | Active | For-profit | Y | 60 | No | None | Male |
| 17 | 230 | Active | Mixed | Y | 25 | Yes | Minor | Male |
| 18 | 94 | Active | NA | Y | 5 | Yes | Minor | Male |
| 19 | 361 | Active | NA | Y | 21 | No | None | Male |
| 20 | 262 | Observation | Non-profit | Y | 9 | No | None | Male |
| 21 | 81 | Active | For-profit | Y | 9 | No | None | Male |
| 22 | 452 | Active | Mixed | Y | 25 | Yes | Minor | Male |
| 23 | 276 | Placebo | For-profit | Y | 21 | No | None | Male |
| 24 | 487 | Active | For-profit | Y | 60 | No | None | Male |
| 25 | 329 | Placebo | For-profit | Y | 44 | No | None | Male |
| 26 | 252 | Active | Non-profit | N | 25 | No | None | Male |
| 27 | 179 | Active | For-profit | Y | 5 | No | None | Female |
| 28 | 658 | Active | For-profit | Y | 60 | No | None | Male |
| 29 | 206 | Active | For-profit | N | 6 | No | None | Male |
| 30 | 94 | Active | NA | N | 4 | No | None | Female |
| 31 | 57 | Active | Non-profit | N | 3 | No | None | Male |
| 32 | 929 | Active | For-profit | Y | 25 | No | None | Male |
| 33 | 792 | Active | For-profit | Y | 60 | No | None | Male |
| 34 | 69 | Placebo | Mixed | Y | 25 | No | None | Male |
| 35 | 124 | Placebo | Mixed | Y | 25 | No | None | Male |
| 36 | 540 | Active | For-profit | Y | 21 | No | None | Male |
| 37 | 70 | Active | Non-profit | N | 21 | Yes | Major | Male |
| 38 | 96 | Active | For-profit | Y | 3 | No | None | Male |
| 39 | 153 | Active | For-profit | Y | 25 | No | None | Male |
| 40 | 256 | Active | For-profit | Y | 25 | No | None | Female |
| 41 | 95 | Placebo | Non-profit | Y | 21 | Yes | Major | Male |
| 42 | 447 | Active | For-profit | Y | 44 | No | None | Male |
| 43 | 220 | Active | for-profit | Y | 9 | No | None | Male |
| 44 | 240 | Active | Non-profit | N | 44 | No | None | Male |
| 45 | 118 | Active | Mixed | Y | 3 | No | None | Male |
| 46 | 39 | Active | Mixed | Y | 12 | No | None | Male |
| 47 | 719 | Placebo | For-profit | Y | 25 | No | None | Female |
| 48 | 625 | Active | For-profit | Y | 9 | No | None | Female |
| 49 | 76 | Active | NA | NA | 2 | Yes | Major | Male |
| 50 | 185 | Active | Non-profit | Y | 9 | No | None | Male |
| 51 | 400 | Active | For-profit | Y | 25 | No | None | Male |
| 52 | 151 | Placebo | For-profit | NA | 3 | No | None | Male |
| 53 | 46 | Active | Mixed | Y | 3 | No | None | Male |
| 54 | 135 | Active | For-profit | Y | 3 | No | None | Male |
| 55 | 254 | Active | For-profit | Y | 9 | Yes | Minor | Male |
| 56 | 54 | Active | For-profit | Y | 5 | No | None | Male |
| 57 | 1579 | Active | Non-profit | N | 21 | No | None | Male |
| 58 | 376 | Active | Non-profit | N | 9 | No | None | Male |
| 59 | 100 | Active | Mixed | N | 3 | No | None | Male |
| 60 | 162 | Active | For-profit | Y | 2 | No | None | Male |
| 61 | 84 | Active | Non-profit | N | 5 | No | None | Female |
| 62 | 800 | Active | For-profit | Y | 25 | No | None | Female |
| 63 | 646 | Active | For-profit | Y | 60 | Yes | Major | Male |
| 64 | 124 | Active | For-profit | Y | 21 | No | None | Female |
| 65 | 1384 | Active | For-profit | Y | 21 | No | None | Male |
| 66 | 224 | Active | For-profit | Y | 2 | No | None | Male |
| 67 | 88 | Active | Non-profit | N | 3 | Yes | Major | Male |
| 68 | 1333 | Active | Non-profit | N | 9 | No | None | Male |
| 69 | 71 | Placebo | Mixed | Y | 6 | Yes | Minor | Male |
| 70 | 302 | Placebo | For-profit | Y | 44 | No | None | Male |
| 71 | 30 | Active | For-profit | NA | 3 | Yes | Major | Male |
| 72 | 126 | Placebo | For-profit | Y | NA | No | None | Male |
| 73 | 2091 | Active | For-profit | N | 44 | No | None | Female |
| 74 | 89 | Active | Non-profit | Y | 3 | No | None | Male |
| 75 | 387 | Active | Non-profit | N | 9 | No | None | Male |
| 76 | 284 | Active | Mixed | Y | 9 | Yes | Major | Male |
| 77 | 164 | Active | For-profit | Y | 12 | No | None | Male |
| 78 | 46 | Placebo | MIxed | N | 6 | No | None | Male |
| 79 | 795 | Active | For-profit | Y | 44 | No | None | Male |
| 80 | 40 | Placebo | Mixed | N | 7 | No | None | Male |
| 81 | 85 | Active | Non-profit | N | 9 | Yes | Minor | Male |
| 82 | 269 | Active | Mixed | Y | 60 | No | None | Male |
| 83 | 280 | Active | For-profit | Y | 44 | No | None | Male |
| 84 | 50 | Placebo | For-profit | N | 4 | No | None | Female |
| 85 | 443 | Active | Mixed | Y | 21 | No | None | Male |
| 86 | 62 | Active | Mixed | N | 6 | No | None | Female |
| 87 | 94 | Active | Mixed | N | 4 | Yes | Minor | Female |
| 88 | 649 | Active | NA | NA | 21 | Yes | Major | Male |
| 89 | 53 | Observation | Mixed | Y | 25 | No | None | Male |
| 90 | 652 | Active | Mixed | Y | 21 | No | None | Female |
| 91 | 37 | Active | For-profit | N | 6 | No | None | Female |
| 92 | 392 | Placebo | For-profit | Y | 60 | No | None | Male |
| 93 | 153 | Active | For-profit | Y | 25 | No | None | Male |
| 94 | 5052 | Active | Non-profit | N | 21 | Yes | Minor | Male |
| 95 | 213 | Active | Non-profit | N | 11 | No | None | Male |
| 96 | 472 | Observation | Mixed | Y | 25 | No | None | Female |
| 97 | 558 | Observation | Mixed | Y | 44 | No | None | Female |
| 98 | 551 | Active | Mixed | N | 25 | No | None | Male |
| 99 | 98 | Active | Mixed | N | 5 | No | None | Male |
| 100 | 105 | Active | Mixed | Y | 9 | No | None | Male |
| 101 | 305 | Active | Mixed | Y | 21 | No | None | Female |
| 102 | 195 | Active | Non-profit | N | 25 | No | None | Male |
| 103 | 54 | Active | For-profit | NA | 2 | No | None | Male |
| 104 | 61 | Active | For-profit | Y | 6 | No | None | Male |
| 105 | 437 | Active | For-profit | Y | 21 | Yes | Minor | Male |
| 106 | 52 | Active | NA | N | 2 | No | None | Female |
| 107 | 1608 | Active | NA | NA | 11 | No | None | Female |
| 108 | 54 | Observation | Mixed | N | 25 | Yes | Major | Male |
| 109 | 128 | Active | Mixed | Y | 9 | No | None | Male |
| 110 | 647 | Active | For-profit | Y | 25 | No | None | Male |
| 111 | 142 | Placebo | For-profit | Y | 60 | No | None | Male |
| 112 | 168 | Active | For-profit | Y | 21 | No | None | Male |
| 113 | 88 | Active | Mixed | N | 4 | No | None | Male |
| 114 | 474 | Observation | For-profit | Y | 25 | Yes | Major | Male |
| 115 | 173 | Active | For-profit | Y | 25 | No | None | Male |
| 116 | 355 | Placebo | For-profit | Y | 25 | No | None | Male |
| 117 | 566 | Active | Non-profit | N | 44 | No | None | Female |
| 118 | 50 | Active | Mixed | Y | 21 | No | None | Female |
| 119 | 113 | Placebo | For-profit | Y | 4 | No | None | Female |
| 120 | 89 | Active | For-profit | Y | 6 | No | None | Male |
| 121 | 521 | Placebo | For-profit | Y | 60 | No | None | Male |
| 122 | 152 | Active | For-profit | Y | 25 | No | None | Male |
| 123 | 2611 | Active | For-profit | Y | 9 | No | None | Female |
| 124 | 74 | Active | Mixed | Y | 25 | No | None | Male |
| 125 | 540 | Active | For-profit | Y | 25 | Yes | Major | Male |
| 126 | 118 | Active | For-profit | NA | 3 | No | None | Male |
| 127 | 140 | Active | For-profit | Y | 6 | No | None | Male |
| 128 | 267 | Active | For-profit | Y | 12 | No | None | Male |
| 129 | 82 | Active | NA | N | 3 | No | None | Female |
| 130 | 50 | Active | For-profit | Y | 4 | No | None | Male |
| 131 | 43 | Observation | Mixed | Y | 3 | Yes | Minor | Female |
| 132 | 100 | Active | For-profit | N | 9 | No | None | Female |
| 133 | 23 | Active | Non-profit | N | 3 | No | None | Female |
| 134 | 101 | Active | For-profit | Y | 3 | No | None | Male |
| 135 | 133 | Active | Mixed | Y | 6 | No | None | Female |
| 136 | 433 | Active | For-profit | Y | 21 | No | None | Female |
| 137 | 110 | Active | For-profit | Y | 3 | No | None | Male |
| 138 | 234 | Active | For-profit | N | 3 | No | None | Male |
| 139 | 380 | Active | For-profit | Y | 21 | No | None | Male |
| 140 | 504 | Active | For-profit | Y | 12 | No | None | Male |
| 141 | 688 | Placebo | For-profit | Y | 21 | No | None | Male |
| 142 | 803 | Active | Non-profit | N | 6 | No | None | Female |
| 143 | 1433 | Active | Mixed | Y | 15 | No | None | Male |
| 144 | 1144 | Placebo | For-profit | Y | 21 | No | None | Male |
| 145 | 361 | Active | For-profit | Y | 5 | No | None | Male |
| 146 | 132 | Active | For-profit | Y | 6 | No | None | Female |
| 147 | 82 | Active | Non-profit | N | 15 | No | None | Male |
| 148 | 89 | Active | Non-profit | N | 15 | No | None | Male |
| 149 | 271 | Active | For-profit | Y | 6 | No | None | Male |
| 150 | 71 | Active | Non-profit | N | 6 | No | None | Female |
| 151 | 127 | Placebo | For-profit | Y | 6 | Yes | Minor | Male |
| 152 | 269 | Placebo | NA | N | 6 | No | None | Male |
| 153 | 80 | Active | For-profit | Y | 6 | Yes | Major | Female |
| 154 | 41 | Active | Non-profit | N | 4 | No | None | Female |
| 155 | 57 | Active | NA | N | 6 | Yes | Minor | Male |
| 156 | 188 | Active | For-profit | N | 4 | No | None | Female |
| 157 | 85 | Active | For-profit | Y | 21 | No | None | Male |
| 158 | 400 | Active | For-profit | Y | 6 | No | None | Male |
| 159 | 122 | Active | For-profit | Y | 7 | No | None | Female |
| 160 | 142 | Active | NA | NA | 4 | Yes | Major | Male |
| 161 | 80 | Active | For-profit | Y | 12 | No | None | Male |
| 162 | 122 | Active | For-profit | NA | 4 | No | None | Male |
| 163 | 109 | Placebo | Mixed | Y | 6 | No | None | Female |
| 164 | 155 | Active | Mixed | Y | 7 | No | None | Female |
| 165 | 345 | Placebo | For-profit | Y | 9 | No | None | Male |
| 166 | 200 | Active | Non-profit | N | 9 | No | None | Male |
| 167 | 90 | Active | Non-profit | N | 4 | No | None | Female |
| 168 | 90 | Active | For-profit | Y | 4 | No | None | Male |
| 169 | 138 | Active | For-profit | Y | 21 | No | None | Male |
| 170 | 80 | Active | Non-profit | N | 9 | No | None | Male |
| 171 | 90 | Active | Non-profit | N | 4 | No | None | Female |
| 172 | 129 | Active | For-profit | Y | 9 | No | None | Male |
| 173 | 116 | Active | Mixed | Y | 9 | No | None | Male |
| 174 | 60 | Active | NA | N | 4 | No | None | Male |
| 175 | 214 | Active | For-profit | Y | 21 | No | None | Male |
| 176 | 83 | Active | For-profit | Y | 4 | No | None | Male |
| 177 | 130 | Active | For-profit | Y | 21 | No | None | Male |
| 178 | 74 | Active | Non-profit | N | 9 | No | None | Male |
| 179 | 147 | Active | Mixed | Y | 9 | No | None | Male |
| 180 | 441 | Active | Non-profit | N | 4 | No | None | Male |
| 181 | 526 | Active | For-profit | Y | 4 | No | None | Male |
| 182 | 107 | Placebo | Non-profit | N | 21 | No | None | Female |
| 183 | 799 | Active | Non-profit | N | 21 | No | None | Female |
| 184 | 142 | Active | NA | N | 3 | Yes | Minor | Male |
| 185 | 97 | Active | Non-profit | N | 21 | Yes | Minor | Male |
| 186 | 75 | Active | Non-profit | N | 9 | No | None | Female |
| 187 | 124 | Placebo | For-profit | Y | 21 | No | None | Male |
| 188 | 72 | Placebo | NA | NA | 21 | Yes | Major | Male |
| 189 | 165 | Placebo | For-profit | Y | 9 | No | None | Male |
| 190 | 131 | Active | For-profit | Y | 3 | No | None | Male |
| 191 | 185 | Placebo | For-profit | Y | 9 | No | None | Female |
| 192 | 365 | Active | For-profit | Y | 9 | Yes | Major | Male |
| 193 | 222 | Active | Mixed | Y | 60 | No | None | Male |
| 194 | 62 | Active | For-profit | N | 6 | No | None | Male |
| 195 | 145 | Active | For-profit | Y | 25 | No | None | Male |
| 196 | 163 | Active | For-profit | Y | 11 | No | None | Female |
| 197 | 159 | Active | For-profit | Y | 15 | Yes | Major | Male |
| 198 | 233 | Active | Non-profit | N | 12 | Yes | Major | Female |
| 199 | 77 | Active | Non-profit | N | 4 | No | None | Male |
| 200 | 481 | Active | Non-profit | N | 9 | No | None | Male |
| 201 | 141 | Active | For-profit | Y | 3 | No | None | Male |
| 202 | 182 | Active | For-profit | Y | 3 | Yes | Major | Male |
| 203 | 1366 | Placebo | For-profit | Y | 25 | No | None | Male |
| 204 | 192 | Observation | Non-profit | N | 3 | No | None | Male |
| 205 | 365 | Active | Mixed | Y | 15 | No | None | Male |
| 206 | 2716 | Active | Mixed | Y | 21 | Yes | Minor | Male |
| 207 | 211 | Active | Mixed | Y | 21 | No | None | Male |
| 208 | 436 | Active | For-profit | Y | 21 | No | None | Male |
| 209 | 64 | Active | Mixed | Y | 9 | No | None | Male |
| 210 | 908 | Observation | Mixed | Y | 21 | Yes | Major | Male |
| 211 | 15 | Active | Non-profit | N | 4 | Yes | Major | Male |
| 212 | 76 | Active | For-profit | Y | 25 | No | None | Male |
| 213 | 230 | Active | For-profit | Y | 25 | No | None | Male |
| 214 | 228 | Placebo | For-profit | Y | 3 | No | None | Female |
| 215 | 175 | Active | Mixed | N | 9 | No | None | Male |
| 216 | 100 | Observation | Mixed | NA | 7 | No | None | Male |
